# Electronic Media Use and Sleep Disorders among Adolescents during the COVID-19 Pandemic

**DOI:** 10.1155/2021/2096944

**Published:** 2021-08-19

**Authors:** Fandi Argiansya, Rismarini Soedjadhi, Raden Muhammad Indra, Yudianita Kesuma

**Affiliations:** ^1^Faculty of Medicine, Universitas Sriwijaya, Palembang, Indonesia; ^2^Department of Pediatrics, Faculty of Medicine, Universitas Sriwijaya and Moh Hoesin Hospital, Palembang, Indonesia

## Abstract

**Background:**

One of the negative impacts of electronic media use is the occurrence of sleep disturbances. Due to the COVID-19 pandemic, the use of electronic media in families, including in adolescents, has been increasing.

**Objective:**

This study was aimed at describing the association between electronic media use and sleep disturbances in adolescents in Palembang.

**Methods:**

A cross-sectional study was conducted in January to February 2021. Participants were 14–17-year-old high school students who completed a questionnaire to assess electronic media use and a Sleep Disturbance Scale for Children (SDSC) questionnaire to assess sleep disturbances.

**Results:**

One hundred and fifty-seven participants enrolled in this study. The majority of the participants were 16 years old or older (56.7%) and used smartphones (93%) with a median of media use of 10 hours a day. None of the participants' characteristic variables showed statistically significant correlations. Similarly, none of the electronic media use variables showed statistically significant correlations.

**Conclusion:**

Most of adolescents in this study have used electronic media for more than 6 years, with median use of 10 hours per day, for noneducative purposes. Despite findings that most of them experience sleep disturbances, there was no statistically significant association between electronic media use and sleep disturbances in adolescents.

## 1. Introduction

Adolescents nowadays are familiar with the use of digital devices. Indonesian children use a variety of digital media at home, such as television, smartphones, video players, radio, gaming equipment, and computers. More than 80% of children aged 10-13 years in Indonesia already have a smartphone. The total daily screen time across all devices has increased from 5 hours/day in 1996 to 8 hours/day in 2016 [1]. Long-term use of electronic media can have various impacts, including physical and psychological health problems such as obesity and sleep disorders.

Sleep disorders in adolescents may be caused by organic or nonorganic factors. Several studies have discussed the relationship between increased screen time intensity and reduced sleep duration and quality [2]. The effects of electronic media use may affect adolescents both positively and negatively. One of the negative impacts is the occurrence of sleep disturbances which are influenced by several factors.

Increasing age indicates an increase in the duration and use of electronic media. Since the start of the COVID-19 pandemic, there has been an increase in the use of electronic media in families due to restrictions on activities outside homes. One study reported that the use of electronic media in children has increased by 87%, while that of their fathers and mothers was 61% and 74%, respectively [3]. According to a study conducted in the USA, there was a significant increase in electronic media use during the pandemic from 32% to 62%, noting that preschool children's use of electronic media has increased from 13% to 26% and school age children's use has also increased from 17% to 44% [4]. The large-scale social restriction policy, a stay-at-home regulation in Indonesia, suspended teaching and learning activities in class as well as group outdoor recreational and/or physical activity, which led to increased use of electronic media. There was no previous study conducted in Palembang, South Sumatra, regarding the impact of electronic media use on the incidence of sleep disorders in adolescents during a pandemic. This study was aimed at describing the association between electronic media use and sleep disturbances in adolescents living in Palembang.

## 2. Methods

A cross-sectional study was conducted in January to February 2021. The participants were high school students of Public Senior High School 17 Palembang aged 14 to 17 years old. All students willing to participate were included. Students who did not complete the questionnaires and students with even numbers on attendance list were excluded. With significance level 5% and study power 20%, a minimum sample size of 118 participants was obtained from a sample size formula. This study was approved by the Hoesin General Hospital Ethical Committee on February 4^th^, 2021 (08/kepkrsmh/2021).

Participants were recruited by stratified random sampling based on an attendance list. After obtaining informed consent, subjects were then given two sets of questionnaires to complete, i.e., a questionnaire to assess the electronic media use and a Sleep Disturbance Scale for Children (SDSC) questionnaire. Questionnaires to assess electronic media utilization elaborated the type of media (televisions, tablets, smartphones, computers, or game consoles), duration of use (normal if ≤10 hours per day, overused if >10 hours per day), and the viewed content (educative or noneducative). The SDSC is a questionnaire that evaluates, along with the total score, 6 different sleep domains (disorder in initiating and maintaining sleep, respiratory disorder, impaired consciousness, sleep-wake transition disorder, somnolent disorders, and hyperhidrosis during sleep). Data analysis was conducted using SPSS for Windows version 23.

## 3. Results

A total of 240 students initially participated; then, 40 students who did not complete the questionnaires and 43 students who met the exclusion criteria were excluded, yielding a final sample of 157 participants. The majority of the participants were ≥16 years old (56.7%) with the oldest being 17 years and 4 months and the youngest being 14 years and 8 months. The mean age of the participants of this study was 16 years and 1 month. The study participants were predominantly female (69.4%). The majority of participants had normal nutritional status (80.3%). Forty-six (29.3%) participants consumed caffeine before sleeping, and 29.3% complained of hearing noises (vehicle noises, animal sounds, snores, television, etc.) while sleeping. The participants' characteristics are illustrated in Table 1.

The majority of participants use smartphones (93%) followed by laptops (75.8%). As many as 140 respondents (89.2%) used electronic media before bedtime. The longest duration of media use was 19 hours, and the shortest was 2 hours, with a median of media use of 10 hours. More than half of the participants have been using electronic media for ≥6 years (52.9%) (Table 2).

A total of 138 (87.9%) participants had experienced sleeping disorders according to SDSC questionnaire results. The prevalence of 6 sleeping disorder domains is illustrated in Table 3.

According to data analysis results, none of the participant's characteristic variables have statistically significant associations (all *p* values were >0.05). Similarly, none of the electronic media use variables showed statistically significant associations (all *p* values were >0.05) (Table 4).

Multivariate analysis of confounding variables was carried out to see if there was a bias in this study. Binary logistic regression analysis was performed, and it was found that there was no significant relationship between each confounding variable and sleep disturbances (Table 5).

## 4. Discussion

This study was designed to find out association between electronic media use and sleep disturbances among adolescents in Palembang. The results showed that all adolescents have been using the electronic media, mostly smartphones, and most of them used it for >10 hours per day. This result is in accordance with a survey in Norway conducted by Hysing et al., which stated that adolescents used smartphones more often than using other electronic media [5]. A study conducted in the USA by Parent et al. found that from 210 adolescents, 27% of them use electronic media >10 hours per day [6].

This study found the prevalence of sleep disorders to be 87.9% in adolescents. It is higher compared to the previous study conducted by Natalita et al. in Bekasi who obtained a prevalence of 62.5% in adolescents experiencing sleep disorders [7]. Possible explanations for this might be that this study was conducted during a pandemic with less physical or recreational activity, more stressful time, and plenty time for electronic media use.

This study found hyperhidrosis as the most common sleeping disorder affecting the adolescents, followed by breathing problems during sleep and sleep-wake transition disorders. This is in contrast to the results of a study on sleep problems in East Jakarta, where the most common types of sleep disorders were sleep-wake transition disorder followed by excessive somnolence and disturbances in initiating and maintaining sleep [8]. Differences in the prevalence and types of sleep disorders may occur due to differences in age of study subjects, study sites, and time of the study. Hyperhidrosis can be caused by several internal and external factors. Internal factors may include feelings of anxiety, stress, and nightmares, while external factors may include room temperature which are influenced by weather, ventilation, and cooling facilities in the room.

Duration of electronic media use was not associated with the incidence of sleep disorder (*p* = 0.605). This is in line with the results of a study conducted by Martin et al. on adolescents who stated that there was no relationship between the duration of electronic media use and sleep duration in adolescents [9].

In this study, the use of electronic media before bedtime and sleep disturbances were also investigated. There was no significant association between the use of electronic media before bed and sleep disturbances experienced by adolescents (*p* = 0.436). Meanwhile, a study conducted by Amalina et al. showed that there was an association between the use of media 30 minutes before bed and sleep disturbances. Possible factors that may explain the difference in study results were the age group (14-17 years old vs. 12-15 years old) and measured activities (media use vs. media use plus other activities) [8].

The viewed content also did not show a statistically significant correlation with sleep disorders (*p* = 0.489). Meanwhile, the study of Martin et al. showed that there was an association between the use of social media at night which could reduce sleep quality and pressure on social media which could have an effect on poor sleep hygiene or difficulty starting to sleep [9]. The difference in study results could be due to the lack of variation in educational programs available.

Overall, the prevalence of sleep disorders in adolescents is increasing; the average duration of electronic media use in a day for teenagers is 10 hours, so that the type of electronic media, program content, onset, and use of electronics before bed no longer affect the prevalence of sleep disorders.

This study is attributed to several limitations that there is no prepandemic data, and results may arise from other variables that are not measured which may affect the significance of the study, such as parenting styles, home environment and hygiene before bedtime, time to use electronic media (morning, noon, and night), and air conditioning facilities used in the room/bedrooms. Sleep disturbances in this study was measured by questionnaires, which may not show objective measurements. Polysomnography (PSG) and wrist actigraphy are the gold standards for the diagnosis of sleep disorders. However, PSG has some weaknesses, including high costs, hospitalization requirements, and the need for an expert to interpret the results. Most importantly, wrist actigraphy is not yet available in Indonesia. Therefore, the authors used the SDSC as a measuring tool for sleep disorders because it is the validated questionnaire capable of detecting sleep disorders and types of sleep disorders experienced by adolescents.

In conclusion, all adolescents in this have been using the media; the most widely used electronic media was smartphones with an average use of 10 hours a day. The majority of the adolescents use electronic media for noneducational programs. There is an increasing prevalence of adolescents experiencing sleep disorders. There are no statistically significant associations found between types of electronic media, the onset of electronic media use, use of electronic media, or program content with sleep disorders in adolescents. Future studies are expected to have a larger sample size with better control over confounding variables.

## Figures and Tables

**Table 1:** Characteristics of study participants (*n* = 157).

Variables	*n*	%
Age		
<16 years old	68	43.3
≥16 years old	89	56.7
Sex		
Female	109	69.4
Male	48	30.6
Nutritional status		
Obese	19	12.1
Overweight	1	0.06
Normal	126	80.3
Undernourished	11	7.0
Caffeine intake before sleeping		
Yes	46	29.3
No	111	70.7
Noises during sleep		
Yes	46	29.3
No	111	70.7

**Table 2:** Electronic media use (*n* = 157).

Variables	*n*	%
Types of electronic medias		
Tablet		
Yes	95	60.5
No	62	39.5
Computers/PCs		
Yes	18	11.5
No	139	88.5
Laptops		
Yes	119	75.8
No	38	24.2
Smartphones		
Yes	146	93.0
No	11	7.0
Television		
Yes	73	46.5
No	84	53.5
Game consoles		
Yes	18	11.5
No	139	88.5
Electronic media use before sleeping		
Yes	140	89.2
No	17	10.8
Duration of electronic media use		
≤10 hours	99	63.1
>10 hours	58	36.9
Content of media		
Educative programs	71	45.2
Noneducative programs	86	54.8
Onset of electronic media use		
≤6 years	83	52.9
>6 years	74	47.1

**Table 3:** Prevalence of 6 sleep disorder domains (*n* = 157).

Variables	*n*	%
Disorders of initiating and maintaining sleep	35	22.3
Sleep breathing disorder	60	38.2
Disorders of arousal nightmares	44	28.0
Sleep-wake transition disorders	58	36.9
Disorders of excessive somnolence	57	36.3
Sleep hyperhidrosis	90	57.3

**Table 4:** Correlation of the participant's characteristics and electronic media use with sleep disorders (*n* = 157).

Variables	Sleep disorders		*p* value	OR (CI 95%)
	Yes	No		
Age				
<16 years old	62 (91.2%)	6 (8.8%)	0.271^∗^	1.768
≥16 years old	76 (85.4%)	13 (14.6%)		(0.193–1.398)
Sex				
Male	41 (85.4%)	7 (14.6%)	0.527^∗^	1.380
Female	97 (89.0%)	12 (11.0%)		(0.507–3.75)
Nutritional status				
Overweight and obese	18 (90%)	2 (10%)	1.000^∗∗^	0.784
Normal & undernourished	120 (87.6%)	17 (12.4%)		(0.167-3.683)
Types of electronic media				
Tablets				
Yes	84 (88.4%)	11 (11.6%)	0.804^∗^	1.131
No	54 (87.1%)	8 (12.9%)		(0.428–2.993)
Computers/PCs				
Yes	17 (94.4%)	1 (5.6%)	0.699^∗∗^	2.529
No	121 (87.1%)	18 (12.9%)		(0.317–20.176)
Laptops				
Yes	103 (86.6%)	16 (13.4%)	0.568^∗∗^	0.552
No	35 (92.1%)	3 (7.9%)		(0.152–2.007)
Smartphones				
Yes	130 (89.0%)	16 (11.0%)	0.132^∗∗^	3.047
No	8 (72.7%)	3 (27.3%)		(0.733–12.667)
Televisions				
Yes	65 (89.0%)	8 (11.0%)	0.682^∗^	1.224
No	73 (86.9%)	11 (13.1%)		(0.464–3.230)
Game consoles				
Yes	16 (88.9%)	2 (11.1%)	1.000^∗∗^	1.115
No	122 (87.8%)	17 (12.2%)		(0.235–5.279)
Use of electronic media before sleeping				
Yes	124 (88.6%)	16 (11.4%)	0.436^∗∗^	1.661
No	14 (82.4%)	3 (17.6%)		(0.430–6.414)
Duration of use				
>10 hours	52 (89.7%)	6 (10.3%)	0.605^∗^	1.310
≤10 hours	86 (86.9%)	13 (13.1%)		(0.469–3.658)
Onset of use				
>6 years	62 (83.8%)	12 (16.2%)	0.136^∗^	0.476
≤6 years	76 (91.6%)	7 (8.4%)		(0.177–1.282)
Content of programs				
Educative	77 (89.5%)	9 (10.5%)	0.489^∗^	1.403
Noneducative	61 (85.9%)	10 (14.1%)		(0.536–3.667)
Caffeine before sleeping				
Yes	41 (89.1%)	5 (10.9%)	0.761^∗^	1.184
No	97 (87.4%)	14 (12.6%)		(0.400–3.501)
Noises				
Yes	43 (93.5%)	3 (6.5%)	0.168^∗^	2.414
No	95 (85.6%)	16 (14.4%)		(0.668–8.723)

**Table 5:** Multivariate analysis of confounding variables.

Step		*B*	SE	Wald	df	Sig.	Exp(*B*)
Step 1^a^	Caffeine before sleeping	0.052	0.569	0.008	1	0.927	1.053
	Noises	0.872	0.662	1.733	1	0.188	2.391
	Obesity	0.043	0.350	0.015	1	0.902	1.044
	Constant	-2.771	0.972	8.122	1	0.004	0.063

Step 2^a^	Caffeine before sleeping	0.880	0.656	1.804	1	0.179	2.412
	Noises						
	Obesity	0.038	0.345	0.012	1	0.912	1.039
	Constant	-2.732	0.868	9.912	1	0.002	0.065

Step 3^a^	Caffeine before sleeping	0.881	0.655	1.808	1	0.179	2.414
	Noises						
	Constant	-2.663	0.597	19.881	1	0.000	0.070

Step 4^a^	Constant	-1.983	0.245	65.659	1	0.000	0.138

## Data Availability

The data are available at https://data.mendeley.com/datasets/6jwppvxwrr/3.
